# The efficacy and roles of combining temozolomide with whole brain radiotherapy in protection neurocognitive function and improvement quality of life of non-small-cell lung cancer patients with brain metastases

**DOI:** 10.1186/s12885-016-3017-3

**Published:** 2017-01-10

**Authors:** Xia Deng, Zhen Zheng, Baochai Lin, Huafang Su, Hanbin Chen, Shaoran Fei, Zhenghua Fei, Lihao Zhao, Xiance Jin, Cong-Ying Xie

**Affiliations:** 1 Zhejiang Provincial Key Laboratory of Aging and Neurological Disorder Research, Wenzhou, 325000 China; 2 Department of Radiotherapy and Chemotherapy, the First Affiliated Hospital of Wenzhou Medical University, No.2 Fuxue Lane, Wenzhou, 325000 China

**Keywords:** Temozolomide, Non-small-cell lung cancer, Brain metastases, Whole brain radiotherapy, Neurocognitive function, Quality of life

## Abstract

**Background:**

Brain metastasis (BM) is a poor prognostic factor for non-small-cell lung cancer (NSCLC). The efficacy and roles of combining temozolomide (TMZ) with whole brain radiotherapy (WBRT) in protection neurocognitive function (NCF) and improvement quality of life (QOL) were investigated and compared with WBRT alone in the treatment of NSCLC patients with BM.

**Methods:**

A total of 238 NSCLC patients with BM were reviewed and categorized into WBRT plus TMZ (RCT) arm and WBRT alone (RT), respectively. The efficacy was evaluated with Pearson chi-square or Fisher’s exact tests, Log-rank test and Cox proportional hazards model. NCF was assessed by using revised Hopkins Verbal Learning Test (HVLT-R), Controlled Oral Word Association (COWA) test and Trail-making Test (TMT). QOL was assessed by the Functional Assessment of Cancer Treatment-Lung (FACT-L) Chinese version 4.0 questionnaire.

**Results:**

The average intracranial objective response (ORR) and disease control rate (DCR) for all the patients were 26.9 and 95.8%, respectively. The intracranial ORR and DCR for RCT and RT arm were 34.9% vs. 20.2% (*p* = 0.01) and 98.4% vs. 92.7% (*p* = 0.03), respectively. The median intracranial progression-free survival (PFS) and overall survival (OS) of NSCLC patients with BM were 5.2 and 7.3 months, respectively. The median PFS of RCT arm was significantly longer than that of RT arm (5.9 vs. 4.9 months, *p* = 0.002). The median OS of the RCT arm was also slightly longer than that of the RT arm (8.5 vs. 5.9 months), but without statistical significance (*p* = 0.11). Multivariate analysis indicated that TMZ was a significant factor for PFS. Statistically significant differences on NCF and QOL were observed between CRT and RT arms at 5 months. RCT showed a trend of toxicities increase compared with RT, however, the toxicities were tolerable and manageable.

**Conclusions:**

Adding TMZ to WBRT in the treatment of NSCLC patients with BM could improve the intracranial ORR, DCR, and median PFS compared with WBRT alone. Although no remarkable difference on median OS was found, adding TMZ could prevent NCF and QOL from worsening. The side effects increased by adding TMZ, but the difference was not statistical significance and toxicities were well tolerated.

## Background

Lung cancer has become the leading cause of cancer related deaths in worldwide [1]. Brain metastasis (BM) is one of the most common complications in non-small-cell lung cancer (NSCLC) patients with more than 10% patients presented with BM at their first hospital visit [2, 3] and 30–40% patients developed it during the course of disease [4]. Whole brain radiotherapy (WBRT) is the standard treatment strategy for BM. However, the prognosis of patients with BM remains poor after WBRT with a median overall survival (OS) of 4–6 months. The effect of systemic chemotherapy is limited due to the impenetrability of brain blood barrier [5, 6], as reported that several chemotherapy drugs in combination with WBRT failed to improve the survival [7].

For the past few decades researchers have found that some drugs may have a positive effect on the NSCLC with BM [8–10]. Temozolomide (TMZ) is a new oral alkylating agent, which is able to cross the brain blood barrier with demonstrated survival benefit in the treatment of high-grade gliomas when administered concurrently with adjuvant radiotherapy [11]. Studies demonstrated that TMZ could be used against a broad range of cancers in vitro including NSCLC [12–14]. Adding TMZ to WBRT may improve the response rate of NSCLC patients with BM [15–17]. However, the potential neurocognitive risks and the influence on the patients’ living quality of combing TMZ with WBRT were less studied. The purpose of this study is to investigate the survival benefits, neurocognitive function (NCF) and quality of life (QOL) influence of WBRT with or without TMZ in the treatment of NSCLC patients with BM.

## Methods

### Patients

We retrospectively reviewed NSCLC patients with BM treated at the First Affiliated Hospital of Wenzhou Medical University from January 2008 to December 2015. The eligibility criteria for this study were as follows: patients were historically diagnosed with NSCLC and had confirmed BM by magnetic resonance imaging (MRI); had at least one measurable BM with diameter larger than 10 mm; patients had no history of TKI administration; had adequate function of major organs (including cardiac, hepatic, and renal function) and hematologic function (absolute neutrophil ≥ 1.5 × 10^9^/L or platelet count ≥100 × 10^9^/L); had no uncontrolled morbidities (e.g., myocardial infarction in the last 12 months); with Eastern Cooperative Oncology Group performance status ≤3; Treated by WBRT with a prescription of 3 Gy/fraction × 10 fractions.

The exclusion criteria were as follows: patients had small cell or mixed small cell histology; patients had EGFR mutations; without at least one measurable lesion according to the Response Evaluation Criteria in Solid Tumors (RECIST) 1.1; lost to follow-up or died within 1 month after starting the treatment; received prior radiotherapy to the brain or TMZ or targeted drugs. This study was carried out according to ethical standards, national and international guidelines. It was approved by the Institutional Review Board and performed at the 1st Affiliated Hospital of Wenzhou Medical University (IRB#:2015041). Written informed consent was obtained from each patient before treatment.

### Treatment schemes

Patients were divided into WBRT + TMZ (RCT) arm and WBRT (RT) arm, respectively. WBRT was planned with two lateral parallel-opposite conformal beams at a prescription of 30 Gy for 10 fractions with a 6-MV photon beam on an Elekta Synergy® linac (Elekta Ltd, Crawley, UK). WBRT plans were delivered through a record and verify system (MosaiQ® v. 1.60Q3, IMPAC Medical Systems, Inc., Sunnyvale, CA). In the RCT arm, TMZ 75 mg/m^2^/day was administered daily during radiation treatment. After the completion of WBRT, TMZ 100 mg/m^2^ was continued for 14 days and repeated every 28 days until unacceptable toxicity or disease progression for up to six cycles.

### Neurocognitive function and quality of life assessment

NCF was assessed by using revised Hopkins Verbal Learning Test (HVLT-R), Controlled Oral Word Association (COWA) test and Trail-making Test (TMT). The HVLT-R is a learning and memory test, in which the patient was asked to learn and recall a list of 12 words over three trials [18]. The TMT is a measure of graphomotor speed and set-shifting to measure the executive function [19]. The COWA Test provides a relatively quick test of verbal fluency and it is believed to place high demands on executive control processes [20].

QOL was assessed by the Functional Assessment of Cancer Treatment-Lung (FACT-L) Chinese version 4.0 questionnaire, which has 34 items with a 5-point Likert scale [21]. The FACT-L had been shown to be reliable and valid instruments to measure the QOL of Chinese lung cancer patients [22].

### Treatment evaluation and follow-up

The response and progression were evaluated weekly during WBRT. Evaluation included a complete history, neurologic examination, QOL assessment, blood counts, and biochemistry profile. After RT, the evaluation was done monthly for the first 6 month, then every 3 months after. Evaluation included physical examination, neurologic examination, QOL assessment, a complete blood count measurement, liver function test, and chest computed tomography (CT) scan. Brain CT with and without contrast, abdominal CT, or bone scan, as well as MRI if necessary, were performed when there were relevant symptoms in patients.

### Definitions and statistical analyses

Pearson chi-square or Fisher’s exact tests (when there were fewer than 5 expected counts in the contingency table) were used to compare the baseline characteristics between RCT and RT arms. Tumor response was assessed according to the Response Evaluation Criteria in Solid Tumors 1.1. OS was defined as the interval from the date of initiation WBRT to the date of death resulted from NSCLC. Intracranial progression-free survival (PFS) was defined as local disease progression, the appearance of new intracranial lesions or both. Intracranial PFS was calculated from the initiation WBRT and the date of confirming progression or death from intracranial progression (if death occurred within 60 days of the last central nervous system assessment date). If the complete survival time of a patient was impossible to obtain or the disease did not progress, patient’ status was assumed as the last known survival and/or contact date. The baseline neurocognitive status was recorded at the first neurocognitive assessment before the start of BM treatment. Adverse events were graded according to the National Cancer Institute Common Terminology Criteria for Adverse Events (NCI-CTCAE) v3.0.

Intracranial PFS and OS were estimated by Kaplan-Meier method. Differences between groups were compared by the log-rank test. In order to identify risk factors associated with intracranial progression, multivariate analyses were conducted with Cox proportional hazard model. Reliable Change Index was used to categorize the change or improvement on NCF and QOL scores [23]. The Reliable Change Index was derived from the standard error of measurement (SEM) of each test, which is calculated from the test-retest reliability (r) and the standard deviation (SD) of test scores: SEM = SD (1-r) ^1⁄2^. The standard error (SE) of difference was then calculated as: SE _diff_ = [2 (SEM ^2^)] ^1⁄2^. All Reliable Change Index thresholds were rounded to the nearest whole number. Scores in any tests decreased from baseline status and met the Reliable Change Index threshold were categorized as deterioration at a specific time period (e.g. 3 months, 5 months and 7 months). The predictive accuracy of various Cox regression models was quantified by Harrell’s concordance index (C-index), which ranges from 0.5 (no predictive power) to 1 (perfect prediction). Statistical analyses were computed using SPSS (version 17.0, SPSS Inc., Chicago, IL) and the R stats package (R Foundation for Statistical Computing, Vienna, Austria). Tests were two sided and *p* < 0.05 was considered statistically significant.

## Results

### Patients’ characteristics

**Fig. 1:**
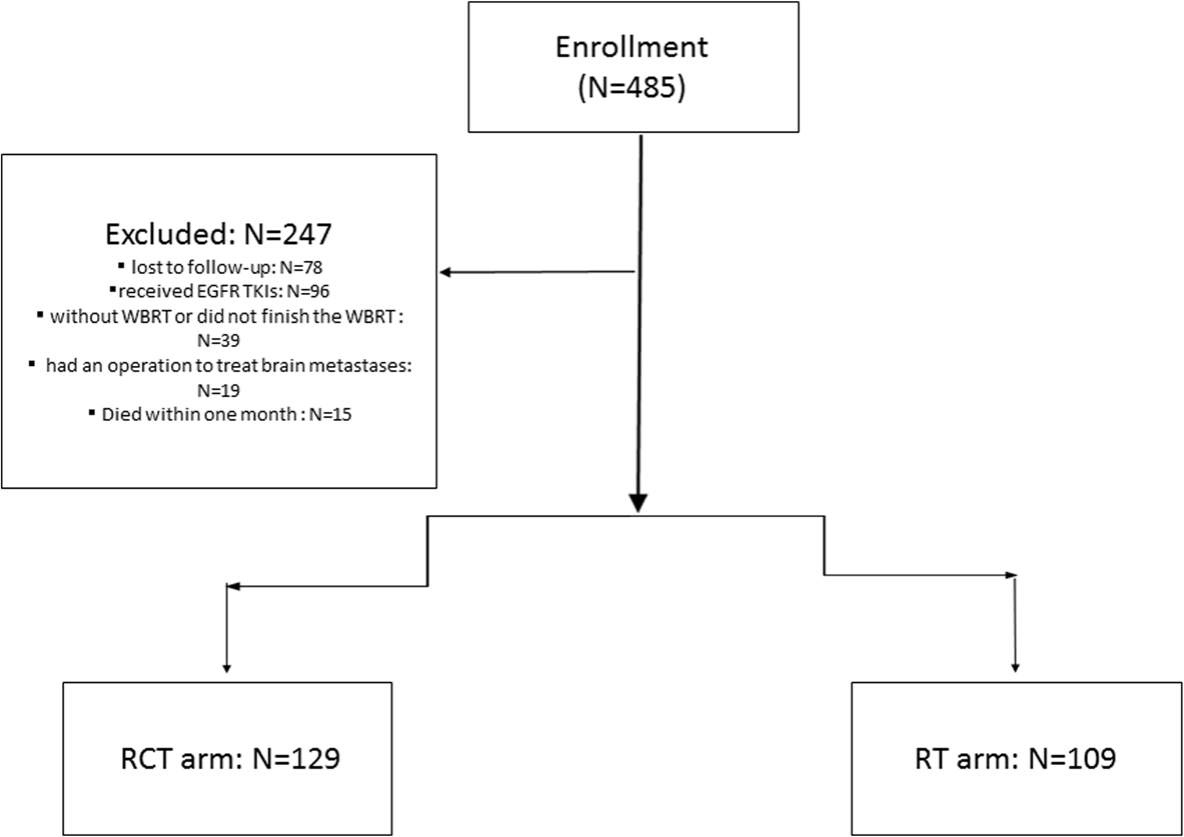
Flow diagram of patients enrollment

From January 2008 to December 2015, 485 NSCLC patients with BM were retrospectively reviewed. Seventy-eight patients due to loss of follow-up information, 96 patients due to received EGFR Tyrosine Kinase Inhibitor (TKIs) and 39 patients due to without WBRT or did not finish the WBRT were excluded. Nineteen patients had an operation to treat brain metastases and 15 patients died within 1 month after starting WBRT were also excluded (Fig. 1). A total of 238 NSCLC patients with BM were enrolled in this study with a median age of 60 years (range, 34–85). There were 129 patients (54.2%) and 109 patients (45.8%) categorized into RCT arm and RT arm, respectively. Baseline characteristics of patients were well balanced between the matched pairs as shown in Table 1.

**Table 1:** Characteristics of NSCLC patients with brain metastasis

Characteristics	Total (%)	RCT (%)	RT (%)	*P*
All patients	238 (100)	129 (100)	109 (100)	
Gender				
Female	102 (42.9)	60 (46.5)	42 (38.5)	0.22
Male	136 (57.1)	69 (53.5)	67 (61.5)	
Smoking				
Never	108 (45.4)	63 (48.8)	45 (41.3)	0.24
Current/former	130 (54.6)	66 (51.2)	64 (58.7)	
Age				
≤60	96 (40.3)	58 (45.0)	38 (35.9)	0.11
>60	142 (59.7)	71 (55.0)	71 (65.1)	
Histology				
Adenocarcinoma	227 (95.4)	123 (95.3)	104 (95.4)	0.98
Non-adenocarcinoma	11 (4.6)	6 (4.7)	5 (4.6)	
ECOG PS				
0–1	188 (79.0)	101 (78.3)	87 (79.8)	0.77
2–3	50 (21.0)	28 (21.7)	22 (20.2)	
Prior chemotherapy				
NO	92 (38.7)	51 (39.5)	41 (37.6)	0.76
YES	146 (61.3)	78 (60.5)	68 (62.4)	
Number of BM				
≤3	65 (27.3%)	33 (25.6%)	32 (29.4%)	0.52
>3	173 (72.7%)	96 (74.4%)	77 (70.6%)	
Extracranial metastases				
NO	93 (39.1%)	48 (37.2%)	45 (41.3%)	0.52
YES	145 (60.9%)	81 (62.8%)	64 (58.7%)	
Primary disease control				
NO	31 (13.0%)	20 (15.5%)	11 (10.1%)	0.22
YES	207 (87.0%)	109 (84.5%)	98 (89.9%)	
RTOG RPA class				
ClassI	50 (21.0%)	31 (24.0%)	19 (17.4%)	0.21
ClassII + III	188 (79.0%)	98 (76.0%)	90 (82.6%)	
RTOG GPA grade				
0–2	143 (60.1%)	74 (57.4%)	69 (63.3%)	0.35
2.5–4	95 (39.9%)	55 (42.6%)	40 (36.7%)	

Abbreviations: Eastern Cooperative Oncology Group performance status *ECOG PS*, Brain metastasis *BM*, the Radiation Therapy Oncology Group *RTOG*, recursive partitioning analysis *RPA*, graded prognostic assessment *GPA*

### Responses and survival of patients

The average intracranial objective response rate (ORR) and disease control rate (DCR) for all patients were 26.9% (64/238) and 95.8% (228/238), respectively. There were 164 patients (68.9%) who had intracranial stable disease and 10 (4.2%) who had intracranial progressive disease. The intracranial ORR for RCT and RT arm were 34.9% (45/129) vs. 20.2% (22/109) (*p* = 0.01), respectively. The intracranial DCR for RCT was 98.4% (127/129) compared with 92.7% (101/129) for RT arm (*p* = 0.03).

**Fig. 2:**
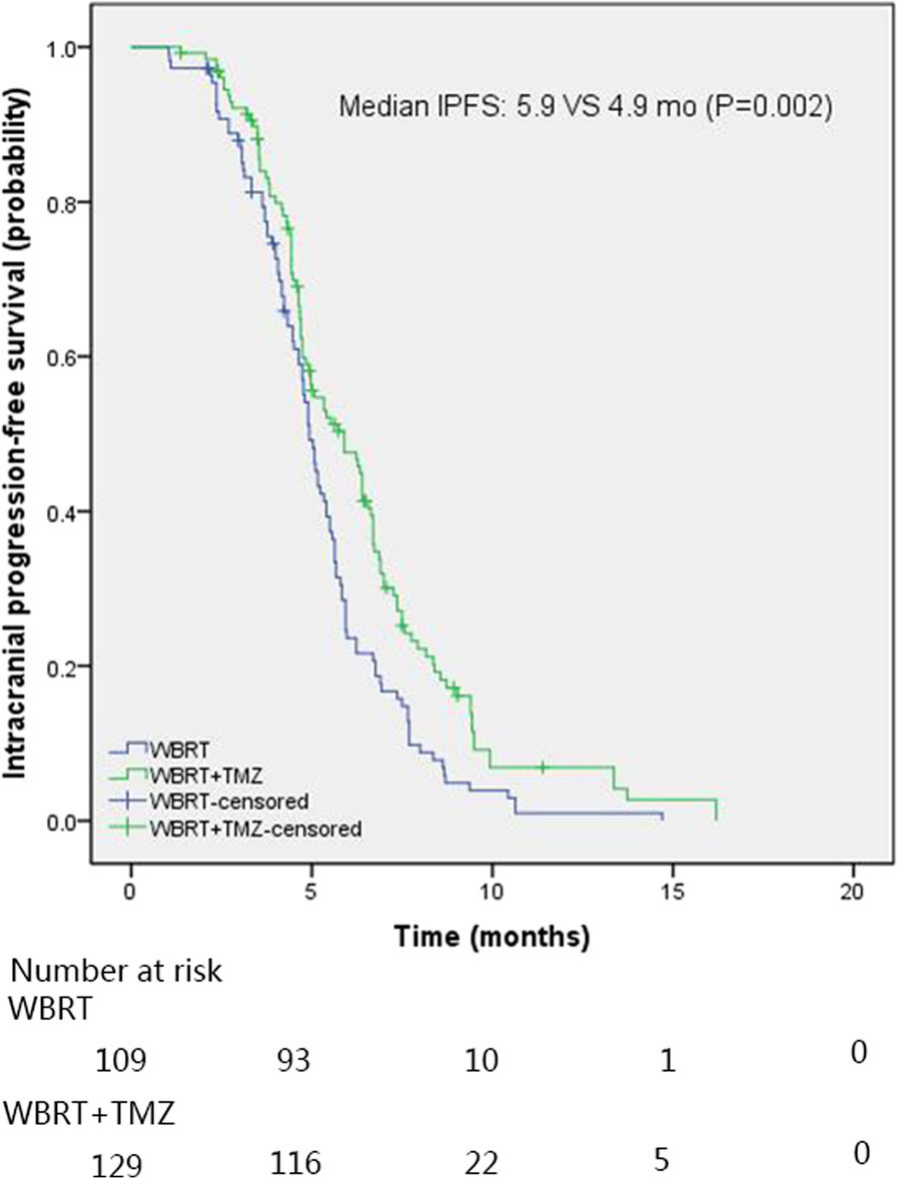
The intracranial progression-free survival of NSCLC patients with brain metastases

**Fig. 3:**
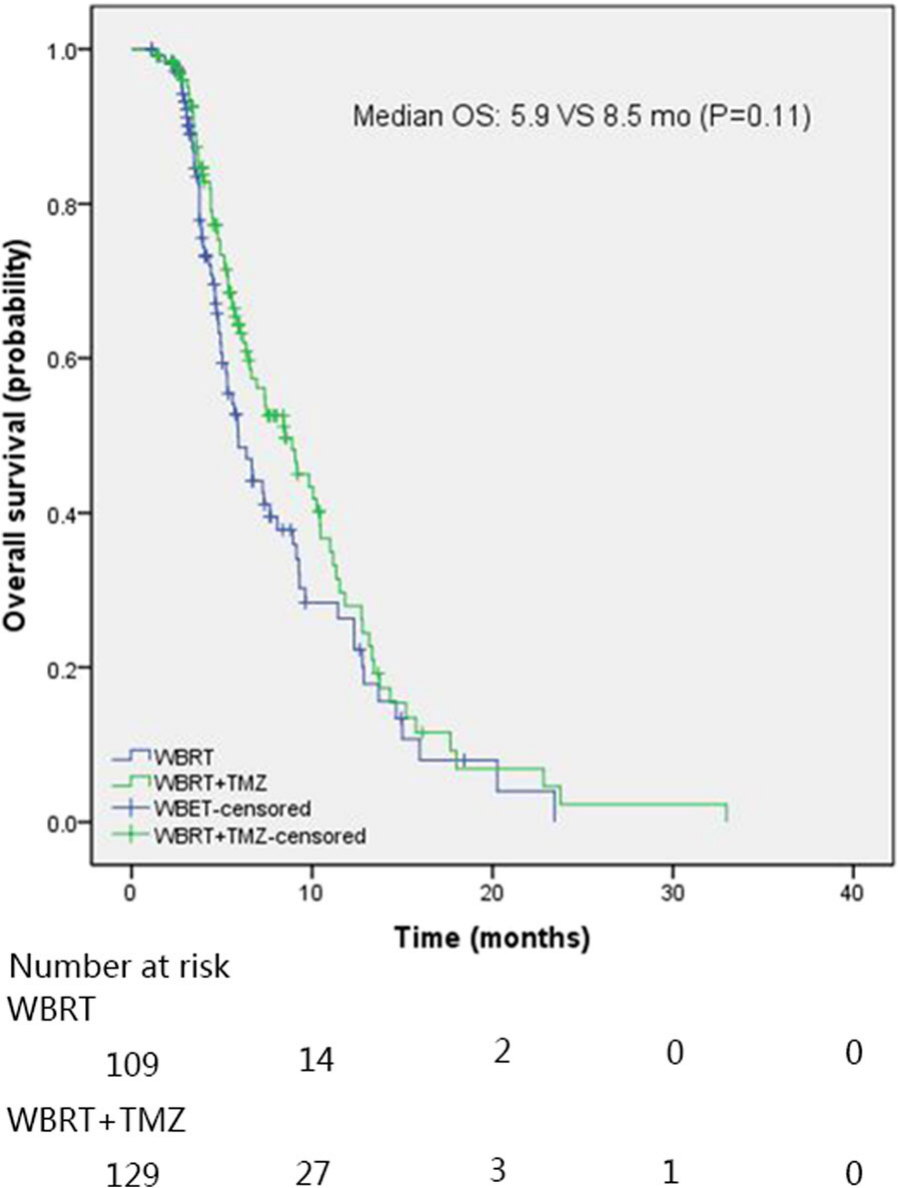
The overall survival of NSCLC patients with brain metastases

The median intracranial PFS and OS for all patients were 5.2 months [95% confidence interval (CI), 4.8–5.6 months] and 7.3 months (95% Cl, 5.9–8.8 months), respectively. The median intracranial PFS of RCT arm was significantly longer than that of RT arm (5.9 vs. 4.9 months, *p* = 0.002) (Fig. 2). The estimated 3-month PFS rates were 92.1% and 87.9% in the RCT arm and RT arm, respectively. The median OS of the RCT arm was slightly higher than that of the RT arm (8.5 vs. 5.9 months) (Fig. 3), but without statistical significance (*p* = 0.11). The estimated 6-month OS rates were 64.3 and 48.4% in the RCT and RT arm, respectively.

Table 2 shows the multivariate analysis results on intracranial PFS and OS for all patients with or without the Radiation Therapy Oncology Group (RTOG) recursive partitioning analysis (RPA) classification and graded prognostic assessment (GPA) grade. For multivariate analysis with RPA and GPA included in the Cox’s regression model, receiving TMZ (*p* = 0.004), never smoking (*p* = 0.02), primary disease controlled (*p* = 0.003) and lower RTOG RPA class (*p* = 0.008) were of prognostic significance for intracranial PFS. The C-index of this model including smoking status, RTOG RPA class, therapeutic schedule and primary disease situation was 0.726 for intracranial PFS; never smoking (*p* = 0.03), lower RTOG RPA class (*p* = 0.01), RTOG GPA grade 2–4 (*p* = 0.02) and primary disease controlled (*p* < 0.001) were correlated with longer OS. The C-index of the model was 0.768 for OS. For multivariate analysis without RPA and GPA entering into Cox’s regression model, TMZ (*p* = 0.004), smoking status (*p* = 0.02), number of BM (*p* = 0.02) and primary disease controlled (*p* = 0.007) were independent prognostic factors for intracranial PFS. The C-index of this model including smoking status, number of BM, therapeutic schedule and primary disease situation was 0.722 for intracranial PFS; smoking status (*p* = 0.03), number of BM (*p* = 0.02), performance status (PS) (*p* = 0.003) and primary disease controlled (*p* < 0.001) were associated with OS. The C-index of the model was 0.758 for OS.

**Table 2:** Multivariate analysis of factors affecting intracranial PFS and OS in NSCLC patients with brain metastasis

Factors	Intracranial PFS				OS		
	N	HR	95%CI	P	HR	95%CI	*P*
With RPA and GPA in the model							
Smoking	108/130	1.42	1.07–1.88	0.02	1.48	1.04–2.10	0.03
Primary disease controlled	31/207	0.54	0.36–0.81	0.003	0.36	0.23–0.57	<0.001
RTOG RPA class	50/188	1.81	1.17–2.79	0.008	1.99	1.15–3.45	0.01
Therapeutic schedule	109/129	0.66	0.50–0.88	0.004			
RTOG GPA grade	143/95				0.58	0.36–0.93	0.02
Without RPA and GPA in the model							
Smoking	108/130	1.39	1.04–1.84	0.02	1.48	1.05–2.10	0.03
Number of BM	65/173	1.47	1.08–2.00	0.02	1.58	1.08–2.33	0.02
Primary disease controlled	31/207	0.57	0.38–0.86	0.007	0.38	0.24–0.60	<0.001
Therapeutic schedule	109/129	0.66	0.50–0.88	0.004			
EGOC PS	188/50				1.76	1.21–2.57	0.003

Abbreviations: *RPA* recursive partitioning analysis, *GPA* graded prognostic assessment, *BM* brain metastasis, Eastern Cooperative Oncology Group performance status *ECOG PS*, the Radiation Therapy Oncology Group *RTOG*, confidence interval *CI*, hazard ratio *HR*

### Comparison of NCF and QOL

Table 3 illustrates the compliance to NCF and QOL assessments at the baseline and over the first 7 months of follow-up. There was no significant difference on the compliance between two arms (*p* > 0.05). Table 4 shows the deterioration status over 7 months as defined by Reliable Change Index threshold baseline. Before treatment, there was no significant difference on the declined number of scores for NCF and QOL between two groups (*p* > 0.05). There were 23 out of 105 evaluated patients from RCT arm deteriorated in HVLT-R delayed recall, which were significant lower than (*p* = 0.02) those in RT arm, in which 32 out of 87 were deteriorated. Statistically significant differences were also found in TMTB (*p* = 0.03) and COWA (*p* = 0.03) at 3 months. For HVLT-R and COWA, there were significantly greater deterioration in HVLT-R total recall (TR) (*p* = 0.008), HVLT-R delayed recall (*p* = 0.007), COWA (*p* = 0.002), FACT-L (*p* = 0.01) in the RT arm compared with RCT arm at 5 months. No statistically significant differences between the two arms was observed at 7 months (*p* > 0 .05).

**Table 3:** Neurocognitive and quality of life assessment compliance

Evaluation Status	RCT arm		RT arm		*P*
	Not evaluated	Received	Not evaluated	Received	
Hopkins Verbal Learning Test (HVLT-R)					
Baseline	5	124	4	105	0.93
At 3 Months	24	105	32	87	0.12
At 5 Months	57	72	55	64	0.75
At 7 Months	87	42	83	36	0.70
Trail-making Test (TMT)					
Baseline	6	123	4	105	0.71
At 3 Months	26	103	31	88	0.27
At 5 Months	59	70	57	62	0.73
At 7 Months	85	44	83	36	0.52
Controlled Oral Word Association (COWA) test					
Baseline	5	124	6	103	0.55
At 3 Months	24	105	30	89	0.21
At 5 Months	57	72	58	61	0.47
At 7 Months	85	44	85	34	0.35
Functional Assessment of Cancer Treatment-Lung (FACT-L)					
Baseline	7	122	9	100	0.48
At 3 Months	27	102	34	85	0.16
At 5 Months	56	73	54	65	0.76
At 7 Months	88	41	86	33	0.49

Abbreviations: Hopkins Verbal Learning Test *HVLT-R*, Trail-making Test *TMT*, Controlled Oral Word Association test *COWA*, Functional Assessment of Cancer Treatment-Lung *FACT-L*

**Table 4:** Deterioration status from baseline in each examination using reliable change index

Deterioration status	RCT arm		RT arm		*p*
	Deterioration	No deterioration	Deterioration	No deterioration	
At 3 months					
HVLT-R TR	21	84	19	68	0.76
HVLT-R DR	23	82	32	55	0.02
TMT Part A	22	81	25	63	0.26
TMT Part B	24	79	33	55	0.03
COWA	19	86	28	61	0.03
FACT-L	23	79	24	61	0.45
At 5 months					
HVLT-R TR	21	511	33	31	0.008
HVLT-R DR	23	49	35	29	0.007
TMT Part A	18	52	25	37	0.07
TMT Part B	21	49	30	32	0.03
COWA	19	53	32	29	0.002
FACT-L	25	48	36	29	0.01
At 7 months					
HVLT-R TR	20	22	19	17	0.65
HVLT-R DR	24	18	17	19	0.38
TMT Part A	23	21	21	15	0.59
TMT Part B	25	19	22	14	0.70
COWA	24	20	20	14	0.71
FACT-L	24	17	21	12	0.66

Abbreviations: *HVLT-R TR* Hopkins Verbal Learning Test total recall, *HVLT-R DR* Hopkins Verbal Learning Test delayed recall, *TMT* Trail-making Test, *COWA* Controlled Oral Word Association, *FACT-L* Functional Assessment of Cancer Treatment-Lung

### Adverse effects

Side effects comparison between RCT and RT arms were presented in Table 5. The most frequent hematologic side effects were anemia (55.9%), neutropenia (52.5%) and thrombocytopenia (47.1%). The most common non-hematologic toxicities were nausea (71.8%), fatigue (62.6%), and vomiting (54.6%). The common grade III/IV toxicity was nausea (20.6%). Neutropenia and nausea were the two most frequent grade III/IV hematologic side effects occurred in RCT and RT arms with a rate of 10.1% vs. 9.2%, and 22.5% vs. 18.3%, respectively. On the whole, all toxicities were generally brief, reversible, and manageable. They were well tolerated after symptomatic treatments.

**Table 5:** Toxicity profile for the NSCLC with brain metastasis patients treated by CRT and RT

Side effects/N (%)	RCT arm (*N* = 129)		RT arm (*N* = 109)		*P* for all grades	*P* for grade III/IV
	All grades	Grade III/IV	All grades	Grade III/IV		
Fatigue	81 (62.8)	16 (12.4)	68 (62.4)	12 (11.0)	0.95	0.74
Anorexia	64 (49.6)	14 (10.9)	47 (43.1)	9 (8.3)	0.29	0.50
Diarrhea	18 (13.9)	0 (0%)	12 (11.0)	0 (0%)	0.50	NA
Nausea	88 (68.2)	29 (22.5)	83 (76.1)	20 (18.3)	0.18	0.43
Vomiting	69 (53.5)	14 (10.9)	61 (56.0)	13 (11.9)	0.70	0.80
Headache	55 (42.6)	13 (10.1)	43 (39.4)	11 (10.1)	0.62	0.99
Anemia	72 (55.8)	5 (3.9)	61 (56.0)	3 (2.8)	0.98	0.91
Neutropenia	66 (51.2)	13 (10.1)	59 (54.1)	10 (9.2)	0.65	0.81
Thrombocytopenia	61 (47.3)	4 (3.1)	51 (46.8)	2 (1.8)	0.94	0.84

## Discussion

The effects and influence on Neurocognitive function and QOL of adding TMZ to WBRT in the treatment of NSCLC with BM were investigated in a total of 238 patients. Our study suggested that TMZ combined with WBRT could significantly enhance the intracranial ORR and DCR, as well as median PFS compared with WBRT alone in the treatment of NSCLC patients with BM, but no remarkable difference on median OS was found. NCF and QOL were also prevented from worsening by adding TMZ.

In this study, the intracranial ORR and DCR of NSCLC patients with BM treated by WBRT + TMZ were 34.9 and 98.4%, respectively, which were significantly higher than 20.2 and 92.7% in the RT arm (both *p* < 0.05). These were consistent with results reported in previous studies that TMZ + WBRT may enhance the overall ORR of NSCLC patients with BM compared with WBRT alone [23, 24]. A multi-institutional trial showed a higher overall ORR (48% vs. 27%, *p* = 0.03) in 103 lung cancer patients with BM treated with TMZ 75 mg/m^2^ per day plus WBRT compared with WBRT alone [24]. Through a meta-analysis, Liao Kai et al. also reported that WBRT + TMZ could significantly improve ORR (risk ratio = 1.55, *p* = 0.003) in the treatment of BM from NSCLC compared with WBRT alone [23]. However, a phase II trial reported that adding TMZ to WBRT did not improve the ORR compared with WBRT alone for 12 chemotherapy-native NSCLC patients with BM [25]. In another phase II trial, for 30 pre-treated recurrent NSCLC patients with BM treated by concurrent WBRT and TMZ (150–200 mg/m^2^/d), only 3 (10) and 6 (20%) patients achieved an objective response and disease control [26]. We inferred that pretreatment influenced the efficacy of TMZ in these phase II patients.

The median OS for all NSCLC patients with BM observed in this study was 7.3 months, which is close to the reported median OS of 8.0 months in the study of Wang Q et al., in which NSCLC patients with BM were treated by WBRT followed by intensity-modulated boost combined with concomitant TMZ [16]. In this study, the median OS and PFS in the WBRT + TMZ group and in the WBRT alone group were 8.5 vs. 5.9 months and 5.9 vs. 4.9 months, respectively. Daniel Chua et al. also demonstrated that WBRT + TMZ had a higher median OS (5.7 vs. 4.4 months) and PFS (3.8 vs. 3.1 months) compared with WBRT alone in the treatment of NSCLC patients with BM [27]. However, their reported median OS and PFS were inferior than ours. We speculated that the difference may resulted from different TMZ doses were administered in two studies. In the study of Daniel Chua, patients received TMZ daily for 21 days, while in our study, TMZ 75 mg/m^2^/day was administered daily during radiation treatment and TMZ 100 mg/m^2^/day was continued for 14 days and repeated every 28 days until unacceptable toxicity or disease progression for up to six cycles.

Previous studies reported that TMZ combing with RT could improve QOL in high grade glioma [28, 29]. A single-institution phase I clinical trial on patients with multiple brain lesions from breast carcinoma treated by capecitabine and TMZ demonstrated that significant improvements in attention span (*p* = 0.047) and emotional function (*p* = 0.016) were observed indicating that adding TMZ was not neurotoxic and may have a beneficial effect [30]. Addeo R et al. also reported that a statistically significant improvement on QOL was found at 3,6 and 9 months for 59 patients treated by 30 Gy WBRT with concomitant TMZ [31]. Similarly, our result implied that adding TMZ in the treatment of NSCLC patients with BM could prevent the NCF and QOL from worsening at 5 months. These studies implied that TMZ as a maintenance therapy may improve patients’ NCF and QOL. This may due to a better intracranial ORR and DCR in RCT group. TMZ may has a certain function of preventing tumor recurrence in brain.

Nausea and fatigue were the most frequent side effects observed for both RCT and RT arms, followed by anemia, vomiting, neutropenia, anorexia and thrombocytopenia, etc. Addition TMZ in the RCT arm showed a trend of increasing the rate of side effects compared with RT alone, as reported in previous studies [27, 32]. However, the difference of the adverse events occurrence between RCT and RT arms was not statistically significant.

One limitation of current study is that it is a retrospective methodology from a single institution experience. The impact of various treatments related outcomes could not be fully evaluated. The number of patients enrolled may not be sufficient enough and the follow-up duration of the study may not be long enough. External validation using other large database for further evaluating the prognostic effect of adding TMZ in the treatment of NSCLC patients with BM would be of great value in clinical practice.

## Conclusion

In a conclusion, adding TMZ to WBRT in the treatment of NSCLC patients with BM could improve the intracranial ORR and DCR, as well as median PFS compared with WBRT alone. However, no remarkable difference on median OS was found. NCF and QOL were also prevented from worsening by adding TMZ. Although the side effects were increased by adding TMZ, the difference was not statistical significance and they were well tolerated.

## Abbreviations

BM: Brain metastasis
CI: Confidence interval
COWA: Controlled oral word association
CT: Computed tomography
DCR: Disease control rate
FACT-L: the Functional assessment of cancer treatment-lung
GPA: Graded prognostic assessment
HVLT-R: Revised Hopkins verbal learning test
MRI: Magnetic resonance imaging
NCF: Neurocognitive function
NSCLC: Non-small-cell lung cancer
ORR: Objective response rate
OS: Overall survival
PFS: Progression-free survival
PS: Performance status
QOL: Quality of life
RPA: Recursive partitioning analysis
RTOG: the Radiation therapy oncology group
TKIs: Tyrosine kinase inhibitor
TMT: Trail-making test
TMZ: Temozolomide
WBRT: Whole brain radiotherapy
